# High prevalence of skin and wound care of hospitalized elderly in Brazil: a prospective observational study

**DOI:** 10.1186/s13104-017-2410-6

**Published:** 2017-02-02

**Authors:** Carleara Ferreira da Rosa Silva, Rosimere Ferreira Santana, Beatriz Guitton Renaud Baptista de Oliveira, Thalita Gomes do Carmo

**Affiliations:** 1 State University of New York at Buffalo, Buffalo, NY USA; 2 Department of Medical-Surgical Nursing, Federal Fluminense University, Niterói, Rio de Janeiro Brazil; 3 Department of Fundamental Nursing, Federal Fluminense University, Niterói, Rio de Janeiro Brazil; 4 Rua Dr. Celestino, 74, 6º andar, Niterói, Rio de Janeiro CEP: 24020-091 Brazil; 5 12 Capen Hall, Buffalo, NY 14260-1660 USA

**Keywords:** Pressure ulcer, Elderly, Wounds and Injuries

## Abstract

**Background:**

Skin changes caused by aging increase the risk of skin damages, such as pressure ulcers, during hospitalization of elderly patients. There is few information about the cost of wound treatment in Brazil. Conversely, skin and wound problems are highly reported among hospitalized elderly patients and caregivers. The purpose is to analyze the socio-demographic and clinical profile associated with skin and wound care in hospitalized elderly.

**Methods:**

This is a prospective observational study. The sample consisted of 75 patients, aged 60 years or more, randomly selected in three hospitals in Rio de Janeiro, Brazil. Data extraction from nursing records of the sample, using cross mapping with Nursing Interventions Classification. Data Synthesis supported by SAS 6.11 (SAS Institute, Inc. Cary North Carolina) in association with SPSS version 14.0 and statistics analysis.

**Results:**

The findings were: age standard deviation 7.8, with minimum as 60, and maximum as 91 years old. Prevalence of women and married seniors. High prevalence of long-term hospitalization. There were 21 Nursing Interventions in the nursing records and seventeen of them related to skin and wound care. They were described in 57 nursing activities, present during 376 evaluations and repeated 1756 times. A significant difference was obtained between age and the presence of the nursing interventions “Positioning” (p-0.004), Eye Care/Hygiene (p- < 0.0001) and Oral Health Maintenance (p-0.0003).

**Conclusion:**

The skin care to prevention and treatment of skin damages represented the major demand of nursing interventions in different clinical conditions of hospitalized elderly.

## Background

The aging process implies in physiological changes in several human tissues and systems. Skin aging is evidenced by impaired tissue response to damages, and diminished moist capacity, resulting in peeling, pruritus and dry skin [1]. Association of skin aging and fragility and chronic diseases, leading causes of hospitalization in elderly, increases the risk for skin damages and poor prognosis, such as infection and death during hospitalization. Therefore, skin health is essential to well-being of the elderly and a central component of nursing care [2].

A major wound problem for the hospitalized elderly is pressure ulcers, affecting approximately three million elderly in the United States in 2006, meaning an overall incidence of 0.4–38% in acute care hospitals [3]. The cost for treating this critical problem was approximately 25 million dollars in 1991, and more than ten years later, the cost remains high, ranging from 37 to 70 million dollars in 2008 [4, 5].

There is few information on the cost of wound treatment in Brazil. Conversely, skin and wound problems are highly reported among hospitalized elderly patients and caregivers. The high variety of products and techniques for skin and wound care seems to influence the development of standardized care in this area for the elderly population.

Moreover, the lack of standardized guideline for skin and wound care elicit the debate about nursing interventions for skin care in the elderly. Nursing Interventions Classification (NIC) [6], as developed and translated into clinical practice in America and other countries, tend to be referential in Brazil. However, few studies focused on the hospitalized elderly. Studies on the wound and skin care in Brazil only include patients currently receiving wound care. As the aging process is global and present in patients in all clinical situations, evaluation of skin aging should be considered in all hospitalized older adults.

The lack of standardized treatment guidelines for the particular population of older adults, alongside with the increased need for care, and intention to disseminate nursing interventions, motivated this research study. The first objective was to describe the socio-demographic and clinical characteristics of the hospitalized elderly. The second one was to identify the nursing interventions according to NIC to skin and wound care in hospitalized elderly. Consequently, this research also verifies the applicability of NIC to skin aging care in elderly patients, describing interventions to both treatment and prevention of damages.

## Methods

This is a prospective observational study. Nursing records of elderly patients from three hospitals in the metropolitan area of Rio de Janeiro, Brazil, were assessed, enclosing one university public hospital and two private hospitals, which are responsible for most of the elderly hospitalization in the mentioned area.

Inclusion criteria were: male and female patients aged 60 years old or more, hospitalized for a minimum of 24 h for clinical or surgical purposes. Patients with incomplete medical/nursing records were excluded from the sample.

The Nursing Interventions Classification (NIC) was chosen to assess nursing records and identify nursing interventions to skin and wound care due to its use and empowerment process in Brazil. The sample size was determined according to some beds and average hospitalization rates of the elderly in the three selected hospitals. Therefore, 40 patients have been chosen from hospital A, 20, from hospital B and 15, from hospital C, totaling a sample of 75 patients. Data were collected from March to August 2012, including the inclusion of new subjects as well as the daily revaluation of included participants until discharge or death.

A Crossmapping between NIC and nursing records was used to identify the nursing interventions to skin and wound care. In the first step, the nursing diagnosis: Impaired Skin Integrity, Impaired Tissue Integrity and Risk of Impaired Skin Integrity were selected for this study, and the correlated nursing interventions from NIC were typed into a Google Docs spreadsheet. The keywords from each intervention were highlighted in the bold and capital letter. In the second step, a blank excel document for each subject was created in the Google Docs file, in which the daily nursing records were entered. The third phase was to use the “locate” option in the nursing records, searching for the keywords from the nursing intervention and mapping the interventions from the sample and the NIC. All interventions identified were plotted to a new file.

After the Crossmapping, all interventions identified were analyzed using SAS 6.11 (SAS Institute, Inc. Cary North Carolina) in association with SPSS version 14.0 and Student’s *t* test. We used Mann–Whitney test for statistical comparisons of clinical and socio-demographic variables between the subgroups. We used Kruskal–Wallis ANOVA test for pairwise comparisons of the variables between three subgroups and the Dunn test for multiple comparisons of the identified nursing interventions. The statistical analysis was processed by the statistical software SAS^®^ System, version 6.11 (SAS Institute, Inc., Cary, North Carolina). The outcomes were summarized with a descriptive analysis using tables expressing the frequency (n) and percentage (%) for the qualitative data. The quantitative data were expressed by average, standard deviation, minimum and maximum for numeric data. This research was approved by a suitably constituted Research Ethical Committee by the protocol 0176.0.2258.000-10 of the institution where the work was conducted and in accordance with the provisions of the Declaration of Helsinki.

## Results

Table 1 presents the socio-demographic and clinical characteristics of the sample. The elderly had mean age of 73.6 years old and discrete prevalence of female subjects. There were 30 (40.0%) bedridden elderly. Long-length hospitalization was prevalent: 49 (65.3%) of the sample. The major clinical problem was cardiovascular disease 67 (89.3%), followed by cancer 24 (32.0%). Regarding cancer incidence, there were five (20.83%) cases of abdominal, four (16.7%) of the colon, four (16.7%) gastric, four (16.7%) prostate, three (12.25%) bladder and one (4.17%) hepatic. Infection was the cause of hospitalization of 12 (16.0%) subjects, described as five (41.7%) pneumonia, two (16.7%) urinary infection, two (16.7%) sepsis, two (16.7%) non- identified infection and one (8.3%) pulmonary sepsis. There was no precise information about infection or sepsis secondary to the hospitalization. Low incidence of pain three (4.0%), depression two (4.0%), prostration one (0.13%), Alzheimer four (5.3%) and anemia three (4.0%) patients as a clinical problem.

**Table 1:** Socio-demographic characteristic of the hospitalized elderly

Variables	N	%
Age in years	73.6 ± 7.8 (60–91)	
Sex		
Female	40	53.3
Length of hospitalization		
More than 10 days	49	65.3
Chronic diseases		
Cardiovascular diseases	67	89.3
Cancer	24	32.0
Secondary diagnoses	13	17.3
Infection disease	12	16.0
Diabetes mellitus	9	12.0
Pulmonary diseases	4	5.3
Genital or urinary diseases	4	5.3
Trauma	3	4.0
Marital status		
Married	37	49.3
Widowed	21	28.0
Single	12	16.0
Divorced	5	6.7
Skin lesions		
Sacral pressure ulcers	9	12.0
Incision	5	6.7
Amputation incision	2	2.7
Bullions lesion	2	2.7
Infected lesion	1	1.3
Cistostomy	1	1.3
Gastrostomy	1	1.3
Back pressure ulcer	1	1.3
Insertion tube wound	1	1.3
Occipital pressure ulcer	1	1.3

Regarding skin and wound care, 32 types of skin damages were identified; seven subjects had more than one skin problem. Fifteen subjects had pressure ulcers (sacrum, ear, occipital and back locations). Two subjects had bulbous lesions, considered as a precursor for pressure ulcers. Therefore, 17 (53.125%) of the skin problems related to pressure ulcers.

The Crossmapping identified 21 Nursing Interventions, in which 17 related to skin and wound care. There were 57 nursing activities within those Nursing Interventions, with 1756 repetitions throughout the 376 re-evaluations during the data collection. Table 2 presents the frequency of mapped interventions corresponding to Essential Nursing Interventions (NIC). This table includes average and standard deviation for the age in years, in the presence or absence of the intervention and the descriptive value (*p* value) from *t* test.

**Table 2:** Relation between the essential nursing interventions mapped and the age of elderly patients

Nursing interventions	n	Presence of the intervention	n	Absence of the intervention	p value^a^
		Average ± SD		Average ± SD	
Positioning	30	76.7 ± 7.6	45	71.6 ± 7.3	0.004
Bathing (at the bathroom)	37	73.1 ± 7.1	38	74.1 ± 8.4	0.58
Eye care	17	80.1 ± 7.1	58	71.7 ± 6.9	<0.0001
Oral health maintenance	24	78.2 ± 7.4	51	71.5 ± 7.1	0.0003
Skin surveillance	58	74.1 ± 7.7	17	72.1 ± 8.2	0.35
Medication maintenance	46	72.6 ± 6.8	29	75.2 ± 9.0	0.15
Bathing (at the bed)	30	74.1 ± 8.3	45	73.3 ± 7.5	0.68
Intravenous therapy	16	72.9 ± 5.9	59	73.8 ± 8.3	0.69
Lower extremity monitoring	18	73.7 ± 8.2	57	73.6 ± 7.7	0.94
Respiration monitoring	18	73.7 ± 8.2	57	73.6 ± 7.7	0.94

*SD* standard deviation

^a^t de Student test

The interventions mapped were Positioning, Bathing (at the bathroom and at the bed), Eye Care, Oral Care, Skin Surveillance, Medication Management, Intravenous Therapy and Lower Extremity Monitoring. There was a significant difference between age and the presence of the nursing interventions “Positioning” (p-0.004), Eye Care/hygiene (p < 0.0001) and Oral Health Maintenance (p-0.0003).

Table 3 presents the relationship between Age and Complementary Nursing Interventions (NIC) where “Diet Staging” (p-0.025) had a significant difference. Table 4 shows one new Nursing Interventions, not described by NIC taxonomy, nevertheless relevant to dermatology and geriatric nursing. Table 5 presents the average and frequency of mapped nursing interventions according to hospitalization length and age.

**Table 3:** Relation between complementary nursing interventions mapped and age

Nursing intervention	n	Presence of the intervention	n	Absence of the intervention	*p * value^*a*^
		Average ± SD		Average ± SD	
Surveillance	54	73.9 ± 8.0	21	72.9 ± 7.3	0.60
Diet staging	58	72.5 ± 7.7	17	77.3 ± 7.2	0.025
Intravenous therapy	40	74.0 ± 6.3	35	73.2 ± 9.3	0.69
Bed rest care	25	71.9 ± 6.3	50	74.5 ± 8.4	0.17
Constipation	46	72.7 ± 7.9	29	75.0 ± 7.6	0.21

*SD* standard deviation

^a^t de Student test

**Table 4:** Related between nursing intervention mapped not described by NIC and age

Nursing intervention	n	Presence of the intervention	n	Absence of the intervention	p value^a^
		Average ± DP		Average ± DP	
Use of diaper management	36	76.2 ± 7.3	39	71.2 ± 7.5	0.004

*SD* standard deviation

^a^t de Student test

**Table 5:** Descriptive analysis of demand of nursing interventions related to age and length of hospitalization

Description	<75 years (n = 42)		≥75 years (n = 33)		p value^a^	<10 days (n = 26)	>10 days (n = 49)	p value^a^
	Ave	Min–max	Ave	Min–max				
Total of valuation	4.5	2–23	3	1–9	0.039	4	4	0.90
Minimum of interventions	3	1–17	6	2–19	0.020	3	5	0.002
Maximum of interventions	8	2–30	10	3–28	0.074	7	11	0.003

*Ave* average, *Min* minimum, *Max* maximum

## Discussion

The mean age in this study is higher than the one from similar studies with hospitalized elderly; however, it seems to be in accordance with the expected growth of older population, as proposed by the United Nations data, which estimate one in five people to be 65 years old by 2035 [7]. Regarding gender distribution, the Brazilian Institute of Geography and Statistics describes a proportion growth of 100 female elderly to 75 male; therefore, the discrete prevalence of female subjects in this sample fits the epidemiologic expectation for the elderly population. The prevalence of married subjects is supported by similar studies in Brazil and the United States, where 57% of hospitalized elderly were married, 7%, divorced, 33%, widowed and 4%, single [8].

The primary causes of long-length hospitalization among elderly in Brazil from 2003 to 2008 were cancer, cardiovascular and pulmonary diseases [9], comparable to the findings in this research. Similarly, the prevalence of long-length hospitalization in this study is substantiated by information provided by Freitas [10], who reported that 20.31% or 2,300,951 elderly had long-term hospitalization in Brazil in 2007.

The low incidence of pain, depression and Alzheimer differ from other studies [11] and elicit the question about misreported information or missing data among the medical records. Conversely, those diagnosis correlated to bedridden elderly in the sample were 40 (30) were bedridden, with complaints of prostration, difficult to mobilization, and higher risk for pressure ulcers.

Association between long-term hospitalization, chronic disease, and aging demonstrates a strong correlation with pressure ulcers and increased the need for nursing interventions in the sample. A study reporting supports this finding that length of hospitalization for the elderly with pressure ulcers is nearly three times longer than hospitalization without those skin lesions in the United States [12].

The incidence of pressure ulcers in the elderly is about 30.8% in Brazil, while it may be about three million adults in the United States [10, 13]. In the sample, 32 types of skin problems were identified, and seven patients had more than one skin problem. There were 15 reports of pressure ulcers including sacrum, ear, occipital and back, and two reports of bulbous skin injuries, considered a precursor of pressure ulcers.

Consequently, more than a half of the skin lesions in the sample were pressure ulcers, a common and painful condition, particularly among the elderly. Skin aging, impaired mobility, and urinary incontinence were factors identified in the subjects with pressure ulcers in this study. This finding is supported by another study with similar sample characteristics [14].

Subjects with 75 years or older had a high correlation between age, skin aging and increased need for nursing interventions. This subgroup also had longer hospitalization length and pressure ulcers. Another study on hospitalized elderly in Brazil found that damage ed skin or tissue integrity management represented half of the nurse plain care, and increased need for skin and wound care as the patient grew older [15, 16].

In our study, there is an increased number of nursing intervention with specific and non-specific skin and wound care, such as positioning, skin surveillance, bed rest care, and lower extremity monitoring, as evidenced by the Crossmapping of nursing activities. Moreover, senior subgroups of subjects received a higher number of nursing interventions, what may emphasize fragility and dependence among the oldest subjects.

This finding reinforces the prominent importance of skin care amongst the elderly, emphasizing that long-term hospitalization increases the risk for pressure ulcers in senior patients. Regardless of the low incidence of wound problems in our sample, the nursing interventions to skin and wound care remained high, indicating a high demand of care for a patient with skin aging.

Essential Nursing Interventions (NIC) [6] had a higher frequency in the crossmapping. Those interventions directly correlate to bedridden elderly patients, with physical impairment, who needed special attention to skin and wound care. Complementary Nursing Interventions (NIC) [6] cross-mapped as Diet Staging and Constipation Management had a higher prevalence among the subgroup aged 75 years old or more. One may infer that aging worsens swallowing capacity in this sample and may cause dysphagia and anorexia. On the other hand, aging may associate to malnutrition in this sample, which is a risk factor for skin problems [17, 18].

The intervention “Diaper Management” is not described by NIC [6]; however, it obtained high frequency in the subgroup aged 75 years old or more. This finding yield nursing activities related to perineal skin care, prevention of dermatitis and pressure ulcers. The new Nursing Intervention “Diaper Management” included attention to time-wise change of the diaper, preventing long skin exposition to diuresis.

The absence of some essential nursing interventions to skin and wound care, such as Pressure Ulcers Care, may be explained by the lower incidence of pressure ulcers in the sample. Contrarily, the presence of 17 nursing interventions for skin and wound care may indicate an effective nurse plan developed to prevent the skin problems in all the patients.

## Conclusion

This study revealed that the majority of nursing interventions for hospitalized elderly focused on skin and wound care, even to elderly without pressure ulcers or another skin impairment. The results demonstrated that older subjects were more dependent, and received more nursing interventions. Some limitations of this study were incomplete nursing records and shortage use of standardized nursing classification.

In summary, the socio-demographic condition yields that the oldest subgroup was fragile, dependent and more exposed to the risk of pressure ulcers, culminating with long-term hospitalizations and more nursing interventions. Facing aging as a reality, this found emphasizes the need for nurses to focus on skin and wounds care in Geriatric settings.

The skin care for the elderly above aesthetics, facing the aging as a risk for lesions, such as pressure ulcers and skin tears, should receive attention. Empowering nurses with competence and knowledge to prevent skin problems s would result in reduced hospitalization length, reduced workload and improved quality of care to the hospitalized elderly. The findings of this research are relevant to the clinical practice of Geriatric and Dermatology Nursing, in particular, we aim to improve the quality of care at our hospital by addressing the challenges presented in this data.
